# Non-communicable disease multimorbidity among tribal older adults in India: evidence from Study on Global AGEing and adult health, 2015

**DOI:** 10.3389/fpubh.2023.1217753

**Published:** 2023-08-24

**Authors:** Abhinav Sinha, Srikanta Kanungo, Debdutta Bhattacharya, Harpreet Kaur, Sanghamitra Pati

**Affiliations:** ^1^ICMR-Regional Medical Research Centre, Bhubaneswar, Odisha, India; ^2^Indian Council of Medical Research, New Delhi, India

**Keywords:** multimorbidity, tribal, older adults, India, SAGE

## Abstract

**Introduction:**

Multimorbidity defined as the simultaneous presence of two or more chronic conditions in an individual is on the rise in low- and middle-income countries such as India. With India aiming to achieve universal health coverage, it is imperative to address the inequalities in accessing healthcare, especially among vulnerable groups such as tribal. Moreover, changing lifestyle has led to the emergence of multimorbidity among tribals in India. We aimed to estimate the prevalence and assess the correlates of multimorbidity among tribal older adults in India.

**Methods:**

We employed nationally representative data from the World Health Organization's Study on Global AGEing and Adult Health conducted in 2015. We included 522 participants aged ≥50 years who reported their caste to be ‘Scheduled Tribe' in the survey. A multivariable regression model assessed the association between multimorbidity and various attributes.

**Results:**

Arthritis, cataract, and hypertension were the most common chronic conditions. The overall prevalence of multimorbidity was ~22.61%. We observed a higher likelihood of having multimorbidity among respondents aged ≥80 years [AOR: 4.08 (1.17–14.18)] than the younger age groups, and among the most affluent group [AOR: 2.64 (1.06–6.56)] than the most deprived class.

**Conclusion:**

The prevalence of multimorbidity among tribal older adults is emerging which cannot be overlooked. Health and wellness centers may be a window of opportunity to provide egalitarian and quality preventive and curative services to achieve universal health coverage. Future studies should explore the outcomes of multimorbidity in terms of healthcare utilization, expenditure, and quality of life in this group.

## Introduction

The escalating burden of non-communicable diseases (NCDs) vis-à-vis prevailing infectious diseases has led to a rise in multimorbidity among low- and middle-income countries (LMICs) such as India (1). Multimorbidity defined as two or more co-occurring long-term conditions may encompass NCDs, a chronic infectious disease, or any mental health condition (2). The growing burden of multimorbidity in India is evident in our recent study which estimated its prevalence to be ~50% among participants aged ≥45 years (3). Multimorbidity is associated with poorer patient-reported outcome measures such as functional ability, psycho-social wellbeing, and health-related quality of life (4–6). Furthermore, the complex care needs in multimorbidity lead to polypharmacy and increased healthcare utilization and expenditure (7–9). Multimorbidity management requires frequent visits to physicians, routine investigations, and long-term use of medicines posing a difficulty in the continuity of care (10).

Social determinants of health are equally relevant for chronic conditions (11). Evidence suggests socioeconomic marginalization increases the health risk of an individual (12). Indigenous people commonly referred to as ‘tribal' are one such vulnerable group that faces disparity in healthcare access (13). Tribal people comprise nearly 8.6% of the total Indian population, most of them live in rural areas with subsistence-based economy (14). Tribal people have distinct socio-cultural beliefs and traditions which include a higher use of alcohol and tobacco making them more prone to several NCDs (15). Additionally, changing lifestyle multimorbidity is emerging as an important public health challenge in this community (16). Most of the data on NCDs among the tribal population are either based on evidence from a single tribe or region, with limited nation-wide studies. However, to achieve universal health coverage, it is pertinent to generate national evidence on NCD multimorbidity among tribal that could help in guiding the existing programs and policies to reduce healthcare inequality in this group. Hence, we aimed to estimate the prevalence of multimorbidity and assess its correlates among tribal older adults in India. This study will generate national evidence on the multimorbidity profile among tribal older adults, which could help the base for designing future policies and interventions.

## Methods

### Overview of data

We employed data from the second round of the World Health Organization's Study on Global AGEing and adult health (SAGE) conducted in 2015. SAGE is a nation-wide survey conducted among a representative sample of older adults aged ≥50 years and a smaller cohort of adults aged 18–49 years. SAGE compiles comprehensive longitudinal data on the health and wellbeing of adults and documents the aging process in six countries: India, China, Russia, Ghana, Mexico, and South Africa. In India, data were collected from six states: Assam, West Bengal, Karnataka, Rajasthan, Uttar Pradesh, and Maharashtra, by adopting a multistage stratified cluster random sampling design to reach the ultimate unit of observation. SAGE utilized community-based face-to-face interviews to collect data through standardized survey instruments. Uniform data collection procedures were followed after training the staff to collect and assimilate the data. The detailed description of methods adopted during the SAGE survey has been documented in the Indian National Report of SAGE, wave-2 (17).

### Study participants

A total of 9116 individuals were surveyed during SAGE, wave-2 (India), out of which 721 individuals reported their caste to be ‘Scheduled Tribe.' Furthermore, in congruence with our objective, we dropped the data of 199 participants aged <50 years, as SAGE provides a representative sample of respondents aged ≥50 years only, whereas participants <50 are a smaller cohort. Thus, a total of 522 participants aged ≥50 years were included in the analysis.

### Outcome variable

The main outcome of interest was multimorbidity (two or more chronic conditions in an individual) generated by a simple sum of all chronic conditions present in an individual, i.e., a chronic disease score. We included a total of ten chronic conditions with nine self-reported chronic conditions: arthritis, stroke, diabetes, chronic lung disease, asthma, depression, hypertension, cataract, and edentulism. Additionally, obesity was calculated by considering weight in kg divided by height in m^2^ using a cutoff for WHO's body mass index (BMI) for South Asian adults (25 kg/m^2^) (18).

### Independent variables

Age was categorized as 50–59 years, 60–69 years, 70–79 years, and ≥80 years. Sex of the participants was reported as male and female. Residence was classified as urban and rural. Education was grouped as “no formal education” and “been to school” on the basis of the question “Have you ever been to school?” Occupation was assessed on the basis of “Have you ever in your life done any type of work (not including housework)?” with the responses as yes or no. Those who responded “yes” were classified as “worked,” while those who said “no” were grouped as ‘never worked.' Partner status was assessed on the basis of “What is your current marital status?” with currently married and cohabiting grouped as ‘have a partner' and never married, separated/divorced, and widowed classified as “no partner.” The wealth index was categorized into five quintiles grouped as the most deprived, 2, 3, 4 and the most affluent. Alcohol consumption was classified as yes or no on the basis of the question “Have you ever consumed a drink that contains alcohol?”

### Statistical analysis

We presented continuous data such as age in mean and standard deviation. Descriptive statistics such as frequency and proportion were used to report the characteristics of the study population and prevalence. All analyses were done utilizing survey weights to compensate for complex survey designs. In the study, a 95% confidence interval (CI) was reported for all weighted proportions as a measure of uncertainty. Bivariate logistic regression assessed the association between multimorbidity and various socio-demographic characteristics, reported as odds ratio (OR) with 95% CI. A multivariable logistic regression model was executed to assess the correlates presented as an adjusted odds ratio (AOR) with 95% CI.

### Ethical considerations

We analyzed anonymous secondary data obtained from IIPS, Mumbai. We requested the data by filling data request form available at: https://iipsindia.ac.in/content/SAGE-data. The data are available after attaching a valid identity proof and data request form. Data set access/downloads are only granted for legitimate academic research purposes. The original SAGE study was approved by WHO's Ethics Review Committee. In India, ethics clearance was also obtained from the International Institute of Population Sciences, Mumbai, which is the partner organization for conducting this study in India.

## Results

The mean age of the participants was observed to be 61.29 ± 8.13 years ranging from 50 to 92 years. Most of the participants (46.17%) were aged between 50 and 59 years. More than half of the respondents were women. In total, 90.8% of the respondents belonged to urban areas (Table 1). Almost two-thirds of the participants had no formal education while nearly one-third of the respondents belonged to the most deprived class.

**Table 1:** Socio-demographic characteristics of the study population, Study on Global AGEing and adult health, 2015 (17).

**Socio-demographic characteristics**	**(*****N** =* **522)**	
	***n*** **(%)**	
Age (years) (*N =* 522)	50–59	241 (46.17)
	60–69	188 (36.02)
	70–79	79 (15.13)
	≥80	14 (2.68)
Sex (*N =* 522)	Male	237 (45.40)
	Female	285 (54.60)
Residence (*N =* 522)	Rural	474 (90.80)
	Urban	48 (9.20)
Education (*N =* 522)	No formal education	325 (62.26)
	Been to school	197 (37.74)
Occupation (*N =* 522)	Never worked	119 (22.80)
	Worked	403 (77.20)
Partner status (*N =* 522)	Have partner	369 (70.69)
	No partner	153 (29.31)
Wealth index (*N =* 522)	Most deprived	157 (30.08)
	2	131 (25.10)
	3	110 (21.07)
	4	63 (12.07)
	Most affluent	61 (11.69)
Alcohol (*N =* 521)	Yes	132 (25.34)
		
	No	389 (74.66)

Arthritis was the most (18.44%) prevalent chronic condition followed by cataract (16.82%) and hypertension (11.80%). The prevalence of obesity was observed to be ~13.28% (Table 2). The overall prevalence of multimorbidity was observed to be 22.61%. A higher prevalence (49.24%) of multimorbidity was observed among respondents aged ≥80 years. Female respondents had a higher prevalence (23.29%) of multimorbidity than their male counterparts. Urban residents (33.19) and participants belonging to the most affluent group (36.89%) had a higher prevalence of multimorbidity (Table 3).

**Table 2:** Profile of chronic conditions, Study on Global AGEing and adult health, 2015 (17).

**Chronic condition**	**Prevalence *n*, % (95% CI)**
Arthritis (*N =* 521)	96, 18.44 (15.18–22.02)
Stroke (*N =* 521)	10, 1.83 (0.09–3.50)
Diabetes (*N =* 520)	32, 6.09 (4.24–8.57)
Chronic lung diseases (*N =* 521)	14, 2.68 (1.47–4.47)
Asthma (*N =* 521)	29, 5.56 (3.75–7.89)
Depression (*N =* 520)	10, 1.84 (0.09–3.51)
Hypertension (*N =* 520)	61, 11.80 (9.09–14.81)
Cataract (*N =* 520)	87, 16.82 (13.62–20.22)
Edentulism (*N =* 521)	61, 11.72 (9.07–14.78)
Obesity (*N =* 488)	65, 13.28 (10.43–16.66)
Multimorbidity (*N =* 522)	118, 22.61 (19.08–26.44)

**Table 3:** Association between multimorbidity and various socio-demographic characteristics, Study on Global AGEing and adult health, 2015 (17).

**Socio-demographic characteristics**		**Multimorbidity present**		
		***n**,* **% (95% CI)**	**Odds Ratio (95% CI)**	**Adjusted Odds Ratio (95% CI)**
**Age (Years)**	50–59	47, 19.50 (14.75–25.17)	Reference	Reference
	60–69	43, 22.81 (17.17–29.70)	1.22 (0.72–2.07)	1.29 (0.73–2.30)
	70–79	21, 26.43 (17.04–37.28)	1.48 (0.76–2.89)	1.61 (0.79–3.27)
	≥80	7, 49.24 (21.26–73.41)	4.00 (1.01–15.96)	4.08 (1.17–14.18)
**Sex**	Male	50, 21.75 (16.44–27.41)	Reference	Reference
	Female	68, 23.29 (18.69–28.75)	1.09 (0.67–1.76)	1.35 (0.70–2.58)
**Residence**	Rural	92, 20.71 (17.08–24.85)	Reference	Reference
	Urban	26, 33.19 (22.75–44.39)	1.90 (0.86–4.17)	1.20 (0.49–2.92)
**Education**	No formal education	63, 19.92 (15.52–24.55)	Reference	Reference
	Been to school	54, 26.84 (20.65–33.24)	1.47 (0.91–2.39)	1.37 (0.81–2.35)
**Occupation**	Never worked	93, 23.14 (19.05–27.50)	Reference	Reference
	Worked	25, 20.82 (14.08–29.43)	1.14 (0.64–2.03)	1.30 (0.71–2.38)
**Partner status**	Have partner	75, 21.02 (16.89–25.61)	Reference	Reference
	No partner	43, 26.03 (19.54–33.45)	1.32 (0.79–2.21)	1.16 (0.65–2.06)
**Wealth index**	Most deprived	24, 15.60 (10.11–22.02)	Reference	Reference
	2	24, 19.52 (13.03–27.84)	1.31 (0.67–2.55)	1.27 (0.65–2.46)
	3	29, 25.56 (17.91–34.73)	1.86 (0.91–3.77)	1.69 (0.85–3.36)
	4	16, 25.29 (15.26–37.94)	1.83 (0.84–3.98)	1.63 (0.73–3.64)
	Most affluent	25, 36.89 (25.39–49.33)	3.16 (1.41–7.07)	2.64 (1.06–6.56)
**Alcohol**	Yes	22, 18.65 (12.17–27.07)	0.73 (0.42–1.28)	0.81 (0.43–1.52)
	No	96, 23.80 (19.69–28.22)	Reference	Reference

The bivariate association showed a significant association of multimorbidity with participants aged ≥80 years and the most affluent class. The multivariable regression model suggested a higher likelihood of having multimorbidity among respondents aged ≥80 years [AOR: 4.08 (1.17–14.18)] than the younger age groups. We observed a higher chance of having multimorbidity among the most affluent group [AOR: 2.64 (1.06–6.56)] than the most deprived class (Table 3).

## Discussion

Although substantial improvement in the healthcare system is being made, the tribal population still remains vulnerable in India (18). The socio-cultural beliefs of tribal groups not only make them distinct from the general population but also expose them to risky health behaviors. NCD multimorbidity is equally relevant for tribal health as the general population since the former group is also witnessing a significant lifestyle change making them more prone to NCD multimorbidity (19). Addressing the healthcare needs of tribal is important to achieve universal health coverage (20); hence, we garnered nationally representative evidence on multimorbidity in this population.

We observed arthritis to be the most commonly prevalent chronic condition followed by cataract and hypertension which is consistent with the findings of a study based on nationally representative data among older adults in India that reported hypertension, gastrointestinal defects, and arthritis to be the most prevalent long-term conditions (6). Nonetheless, a study conducted among *Mishing* tribes of Assam reported that 11% of the respondents had obesity which is in congruence with our findings (21). We observed the prevalence of multimorbidity to be 22.61%, which is higher than the prevalence (14.5%) reported by a previous study based on nationally representative data among older adults (22). A probable reason for this could be that our study included higher-aged participants (≥50 years), whereas Puri et al. considered lower-age group respondents (≥45 years). Moreover, it is well known that multimorbidity increases with an increase in age. However, it is worth noting that the prevalence of multimorbidity among tribal people is lesser than that of the general population as previous studies suggest that the prevalence of multimorbidity among the general population aged ≥45 years is ~50% (3, 7, 8). This further emphasizes that early behavioral change communication (BCC) for lifestyle modification such as physical exercise, low salt intake, and abstaining from alcohol and tobacco may be crucial in preventing a further increase in the burden of NCD multimorbidity among tribal groups. Culturally appropriate and linguistically tailored health messages through each tribe's folklore, puppet show, dance, nukkad natika, etc. may be beneficial in reaching the masses. Nonetheless, rather than individuals, families should be the center of intervention.

We observed higher chances of having multimorbidity among older adults aged ≥80 years and above, which is in congruence with the findings of previous studies which suggest that multimorbidity increases with an increase in age (23). The chronic conditions start to appear in midlife at 40–45 years of age and tend to accumulate with an increase in age (6), which could have been one of the reasons for a significant association of multimorbidity among adults aged 80 years and above in the present study. Nonetheless, this age group requires special attention as they tend to be more socially and economically dependent on the younger age groups for treatment. Additionally, in deprived sections of society, the health of these individuals is often neglected as the priority shifts more toward the earning member of the family. Telemedicine services may provide a continuum of care and reduce physical visits to physicians.

We found a significant association between the most affluent class and multimorbidity which is consistent with the findings of a study conducted among 11,365 tribal older adults in India (22). Since our findings are based on self-reported chronic conditions, a probable reason for the affluent class to have more multimorbidity could be due to a better diagnosis of these conditions as compared to the deprived group. The affluent class has a higher capacity to pay, and hence, they may easily avail of healthcare services leading to better diagnosis of conditions. Health and wellness centers envisioned with providing preventive and curative services for chronic conditions may act as a window of opportunity to provide egalitarian and accessible primary care services for all. Nonetheless, to make universal health coverage a reality, we need to especially focus on the marginalized sections of society. The findings of this study highlight two main points: (1) multimorbidity is equally prevalent among tribal older adults, (2) multimorbidity is associated with affluence which highlights that the affluent group might be able to afford healthcare services due to which they were better diagnosed and, hence, points toward equitable healthcare systems that could be accessible and affordable for all strata of society so as to achieve UHC. Additionally, in LMICs such as India which is witnessing an epidemiological transition, it is common for the affluent groups to have NCDs, which could be attributed to lifestyle-related factors such as sedentary habits and consumption of high-fat food. The newly formed cadre of community health officers may help in BCC activities which would help in lifestyle modifications.

### Strengths and limitations

This study is based on a nationally representative sample; however, it is limited by the use of self-reported chronic conditions that may undermine the true population prevalence. Additionally, we used cross-sectional data that could not establish causality.

## Conclusion

The prevalence of multimorbidity among tribal groups is rising and cannot be overlooked. Older age and affluent groups within the tribal population need to be kept at the center of behavioral change communication for lifestyle modifications. Health and wellness centers may be an opportunity to provide egalitarian, accessible, and quality preventive and curative services for this group. Future studies should explore the impact of multimorbidity on healthcare utilization and expenditure of tribal people.

## Data availability statement

Publicly available datasets were analyzed in this study. This data can be found at: https://iipsindia.ac.in/content/SAGE-data.

## Ethics statement

Ethical approval was not required for the study involving humans in accordance with the local legislation and institutional requirements. Written informed consent to participate in this study was not required from the participants or the participants' legal guardians/next of kin in accordance with the national legislation and the institutional requirements. No potentially identifiable images or data are presented in this study.

## Author contributions

Concept, design, and drafting of the manuscript: AS and SP. Acquisition, analysis, or interpretation of data: AS, SK, DB, HK, and SP. Critical revision of the manuscript for important intellectual content: SK, DB, and HK. Statistical analysis: AS. Administrative and technical support: SK, DB, and SP. Supervision: SP. All authors contributed to the article and approved the submitted version.
